# An Account of the Good Effects of Mercury in a Disease Apparently of the Lymphatic System, Attended with Nervous Symptoms

**Published:** 1787

**Authors:** John Covey

**Affiliations:** Apothecary at Basingstoke, in Hampshire


V. *An Account of the good Effects of Mercury in a Difeafe apparently of the Lymphatic Syftem, attended with nervous Symptoms. Communicated in a Letter to Dr.* Simmons, *by Mr.* John Covey, *Apothecary at Bafingftoke, in Hampfhire.*

*To Dr.* SIMMONS.

SIR,

THE following cafe, which fell under my care fome few years ago, having been attended with very fingular as well as extraordinary circumftances, I have a defire, through the channel of the Medical Journal, to communicate it to the public. Dr. Adair faw the patient on the 18th of March; and although he did not afterwards fee her, yet he was regularly informed informed by me of the progrefs of the illnefs, and prefcribed for the patient by letter until her recovery. I have informed him of my intention, and it is by his authority I have made ufe of his name. If you, Sir, fhall judge the cafe fufficiently interefting for publication, you will oblige me by inferting it.

I take this opportunity of requefting you to rectify a miftake in the printing of my laft paper.—The words, "fimilar to thofe," introduced in the third and fourth lines, page 17, of that paper, were not a part of my obfervations; this, together with the fubfequent fentences, being intended only as ftating cafes related by Dr. Monro, Dr. Rufh, and Mr. Quier, which had efcaped my recollection when I penned my firft obfervations on inoculation, and not as ftating facts which had happened under my own infpection *.

I am, Sir,

Your moft obedient fervant,

JOHN COVEY.

*Bafingftoke,*

April 5, 1787.

D. A., a ftrong, healthy girl, aged eight years, and born of healthy parents, was attacked

* See page 17 of the prefent volume.

tacked with the meafles about Chriftmas. The difeafe went off without medical affiftance, and her mother afterwards gave her feven or eight dofes of ftrong fenna tea, which operated very brifkly. About fix weeks after this courfe of phyfic, fhe was attacked with a violent colic, which, after two or three hours continuance, went off. From this time till the Chriftmas following fhe had a return of the colic once in five or fix weeks; and from that time till the beginning of March following the returns were more frequent, generally attacking her once in a week or ten days. About the beginning of that month her mother perceived fome alteration in her fpeech, and that fhe at times complained of pains and ftiffnefs in her knees and elbows. On the 9th I was fent for, and found her with a confiderable degree of fever, and a rafh, refembling the fting of nettles, over the greateft part of her body. This kind of rafh, I was informed, had appeared upon her feveral times in the courfe of the preceding year; but as it was attended with a flight degree of illnefs only, and foon difappeared, but little notice had been taken of it. Both the rafh and the violence of the fever were removed in the course of two or three days, when she again began to complain of pain in her joints, faultered very much in her speech, and had frequently such gestures as are usual in the Chorea sancti Viti; the little fever she now had appearing in the evening only. On examining the pained parts, small moveable knots were observed, which, on being pressed, were somewhat painful. She generally awaked two or three times each night, apparently in violent pain, crying very much; and on being asked where she was in pain, she mentioned sometimes one joint and sometimes another. These complaints so greatly increased, that, at the end of four weeks, she lost her speech, all parts of her frame were almost constantly in motion, and the knots appeared on nearly every joint, and likewise on some other parts of her body, particularly on the whole length of the spine, on her shoulders, round the scapulæ, on the sternum, the elbows, wrists, knuckles, hips, knees, and ancles. Those on the spine, round the scapulæ, and on the knees and breast, were some of them as large as chesnuts; the others from the size of vetches to that of horsebeans. Those upon her breast, and round the scapulæ, appeared to be somewhat inflamed; the others were of the natural colour of the ſkin. The raſh frequently appeared on different parts, but ſoon diſappeared again, leaving a purple efflo-reſcence on the parts for a ſhort time after it was gone. She had at this time quite loſt the uſe of her legs and arms, and awaked many times in the night, apparently in conſiderable agony. If her arms or legs were moved from the poſture they at any time lay in, ſhe expreſ-ſed great ſign of pain, and they were bent with much difficulty.

During the former part of this time ſome medicines, chiefly thoſe called nervous, were tried, but without effect. On the 18th of March Dr. Makittrick Adair, now of Bath, ſaw her; and as there was ſome reaſon to ſuſpect that worms, or ſome other irritating cauſe in the firſt paſſages, might create or ſupport the nervous ſymptoms, he ordered her to take four grains of calomel two nights ſucceſſively, and on the ſecond morning twelve grains of jalap in powder. No relief, however, was obtained from theſe medicines. The warm bath (aſſiſted by ſudorifics) was afterwards tried every day till the 8th of April. Some other remedies of the alterative and antiſpaſmodic kinds were like-wiſe adminiſtered; but as theſe were equally inefficacious, inefficacious, it ſeems unneceſſary to deſcribe them particularly.

On the 9th of April the Doctor directed a ſmall quantity of mercurial ointment to be rubbed into her ſhins at bedtime, and to be repeated each night till it ſhould produce a tenderneſs in the gums, and then it was to be purged off; the warm bath to be continued. From this time the diſorder appeared to be at a ſtand, and on the 16th ſhe ſpoke two or three words, got on her legs, and ſtood up a ſhort time with very little aſſiſtance. The knots now appeared to be rather decreaſing in their ſize, and the nervous ſymptoms were leſs violent. This mercurial courſe was perſiſted in about a month, during which time the gums were only ſlightly affected; the knots and nervous affections gradually decreaſed, and ſhe ſoon after this time regained perfect health. During the whole of this illneſs ſhe had a good appetite, was regular in her body, and although ſhe loſt her ſpeech, yet her ſenſes appeared to be unimpaired.

